# Risk Factors, Patterns, and Long-Term Survival of Recurrence After Radiofrequency Ablation With or Without Transarterial Chemoembolization for Hepatocellular Carcinoma

**DOI:** 10.3389/fonc.2021.638428

**Published:** 2021-05-27

**Authors:** Jingjun Huang, Wensou Huang, Yongjian Guo, Mingyue Cai, Jingwen Zhou, Liteng Lin, Kangshun Zhu

**Affiliations:** Department of Minimally Invasive Interventional Radiology, The Second Affiliated Hospital of Guangzhou Medical University, Guangzhou, China

**Keywords:** hepatocellular carcinoma, radiofrequency ablation, recurrence, risk factor, survival

## Abstract

**Objectives:**

To classify hepatocellular carcinoma (HCC) recurrence patterns after radiofrequency ablation (RFA) or transarterial chemoembolization (TACE) combined with RFA (TACE-RFA) and analyze their risk factors and impacts on survival.

**Methods:**

We retrospectively evaluated the medical records of HCC patients who underwent RFA or TACE-RFA from January 2006 to December 2016. HCC recurrences were classified into four patterns: local tumor progression (LTP), intra-segmental recurrence, extra-segmental recurrence, and aggressive recurrence. Risk factors, overall survival (OS), and post-recurrence survival of each pattern were evaluated.

**Results:**

A total of 249 patients with a single, hepatitis-B virus (HBV)-related HCC ≤ 5.0 cm who underwent RFA (HCC ≤ 3.0 cm) or TACE-RFA (HCC of 3.1-5.0 cm) were included. During follow-up (median, 53 months), 163 patients experienced HCC recurrence: 40, 43, 62 and 18 patients developed LTP, intra-segmental recurrence, extra-segmental recurrence, and aggressive recurrence, respectively; the median post-recurrence survival was 49, 37, 25 and 15 months, respectively (P < .001); the median OS was 65, 56, 58 and 28 months, respectively (P < .001). Independent risk factors for each pattern were as follows: tumor sized 2.1-3.0 cm undergoing RFA alone and insufficient ablative margin for LTP, periportal tumor and non-smooth tumor margin for intra-segmental recurrence, HBV-DNA ≥ 2000 IU/mL for extra-segmental recurrence, and periportal tumor and α-fetoprotein ≥ 100 ng/mL for aggressive recurrence. Recurrence pattern (P < .001) and Child-Pugh class B (P = .025) were independent predictors for OS.

**Conclusions:**

Based on our classification, each recurrence pattern had different recurrence risk factors, OS, and post-recurrence survival.

## Introduction

Hepatocellular carcinoma (HCC) is the fifth most common cancer and the second most frequent cause of cancer-related death globally (1). Radiofrequency ablation (RFA) is the preferred treatment approach for hepatocellular carcinoma (HCC) patients who are not amenable to surgery or liver transplantation (1). RFA has been considered as a curative treatment in patients with small HCC that is 3.0 cm or less in diameter (2–4). However, the 5-year recurrence rate still reached 65.6%-69.8% (5, 6), with the 5-year overall survival (OS) rate of 55.2%-65.1% (3, 7, 8). For patients with intermediate-sized tumor that is between 3.1 cm and 5.0 cm in diameter, although results from randomized trials indicate the survival benefit associated with the combination approach with transarterial chemoembolization (TACE) and RFA (TACE-RFA) compared with RFA alone (9–11), the 3-year local tumor progression (LTP) rate still reached 28%-40% (9, 10). HCC recurrence after RFA or TACE-RFA is very common, which limits the improvement of long-term survival.

The mechanisms and risk factors for HCC recurrence after RFA are various and difficult to identify and quantify. First, it is important to consider the inherent technical factors related to the RFA procedure, such as tumor dissemination or metastases caused by heating because of increased intra-tumoral pressure during the RFA (12) or needle-track tumor seeding by the puncture (13). A study (14) suggested that certain residual HCC cells (incomplete ablation or insufficient ablative margin) may transform into a highly proliferative and more aggressive cellular phenotype after going through sublethal temperatures, which accelerates HCC recurrence or metastases. The second consideration is multicentric hepatocarcinogenesis. Chronic viral hepatitis leads to repeated destruction and regeneration of liver parenchyma, which induces the accumulation of mitochondrial DNA mutations (15) and *de novo* carcinogenesis. Third, oncologic factors such as large tumor size (16, 17), non-smooth tumor margin (18), and high α-fetoprotein level (19), may indicate that the HCC has a higher malignant degree and a higher risk of tumor invasion and HCC recurrence. Fourth, HCC at a perivascular location, subphrenic location, or location adjacent to the colon may increase the difficulty of complete ablation and have the risk of tumor dissemination or spread through the portal or hepatic vein system (13, 16, 17, 20).

The various mechanisms and risk factors may lead to different patterns of HCC recurrence after RFA or TACE-RFA, such as LTP, intra-segmental recurrence, extra-segmental recurrence, extrahepatic metastasis, portal vein or hepatic vein invasion, needle-track tumor seeding, or even multiple coexisting recurrence patterns. And, these recurrence patterns may carry different prognostic value in relation to long-term survival. Screening these risk factors and identifying the influence of different recurrence patterns on long-term survival may carry crucial clinical implications. However, currently, studies (21, 22) reported on risk factors for different recurrence patterns after RFA or TACE-RFA were less frequent, and the follow-up was not long enough for analysis of long-term survival. Therefore, the purpose of this study was to classify HCC recurrence patterns after RFA or TACE-RFA and analyze their risk factors and impacts on long-term survival.

## Materials and Methods

### Patients

We retrospectively evaluated the medical records of patients with HCC who underwent RFA or TACE-RFA from January 2006 to December 2016 at our institution. Patients with HCC of 3.1-5.0 cm underwent TACE-RFA, and patients with HCC of ≤ 3.0 cm underwent RFA alone, since TACE-RFA, compared to RFA alone, was reported to improve local tumor control and survival for HCC of 3.1-5.0 cm (9, 23), but not for HCC of ≤ 3.0 cm (24, 25). The diagnosis of HCC was confirmed according to the European Association for the Study of Liver/American Association for the Study of Liver Disease guidelines (26). Patients were included in our study if they: (a) were aged 18 to 75 years; (b) had an Eastern Cooperative Oncology Group (ECOG) performance status of 0-1, (c) had Child-Pugh class A or B liver disease; (d) had one HCC mass ≤ 5.0 cm in diameter who achieved complete ablation based on the contrast-enhanced computed tomographic (CT) or magnetic resonance (MR) images one month after RFA or TACE-RFA treatment, (e) had positive test for hepatitis B surface antigen and negative test for antibodies to hepatitis C virus, and received a regular antiviral therapy with nucleotide analogues initiated before or after treatment, (f) were unwilling to undergo hepatectomy or liver transplantation, (g) had no evidence of invasion into the portal or hepatic venous branches based on CT or MR images, and (h) had no evidence of extrahepatic metastases. Patients were excluded from this study if they: (a) had previously undergone hepatic resection or locoregional therapies (TACE, RFA, microwave ablation or percutaneous ethanol injection) before RFA or TACE-RFA, (b) had current or a history of malignant tumors in addition to HCC, (c) severe medical comorbidities including severe dysfunction of the heart or kidney and untreatable/unmanageable severe coagulation disorders (prothrombin time ≥ 18 seconds or a platelet count of < 30 × 10^9^/L), and (d) had a follow-up less than six months. This retrospective study was approved by our institutional review board. Written informed consent was obtained from every patient before treatment.

### TACE Procedure

TACE was performed by one of four physicians. TACE was performed by placing a 5-F catheter (Cook, Bloomington, Indiana) or a 2.8-F microcatheter (Renegade Hi-Flo Straight, Boston Scientific, Natick, Mass; Progreat, Terumo, Tokyo, Japan) as superselectively as possible into tumor-feeding arteries. Initially, a lobaplatin solution with a concentration of 0.5 mg/mL was infused into the tumor-feeding vessels. The total lobaplatin level was 20-50 mg and depended on the patient’s body weight. Then, an emulsion of 3–10 mL lipiodol (Lipiodol Ultrafluide; Guerbet, Aulnay-Sous-Bois, France) and 20–60 mg doxorubicin hydrochloride was administered into the feeder vessels. Finally, polyvinyl alcohol particles 300 μm in diameter (Polyvinyl Alcohol Foam Embolization Particles; Cook) mixed with contrast material were administered into the tumor-feeding vessels until arterial flow stasis was achieved. After embolization, angiography of the feeding artery was performed to determine the extent of vascular occlusion. If the feeding artery was not completely occluded, polyvinyl alcohol particle embolism was performed again.

### RFA Procedure

RFA was performed percutaneously under CT guidance within 1–4 weeks after TACE, while the patient was under conscious sedation and local anesthesia. RFA procedures were performed by one of four physicians with at least 5 years of experience performing CT-guided percutaneous RFA. We used an RFA system (RITA 1500X RF generator, RITA Medical Systems; AngioDynamics, Manchester, Ga). For tumor larger than 2.0 cm, a 14-gauge electrode needle with nine expandable electrode tines (StarBurst XL, RITA Medical System; AngioDynamics) was used, which can produce a necrosis area with a maximum diameter of 3.1–5.0 cm. For tumor no larger than 2.0 cm, a 17-gauge electrode needle with three expandable electrode tines (StarBurst SDE, RITA Medical System; AngioDynamics) was used, which can produce a necrosis area with a maximum diameter of 2.0 cm. All treatments were performed according to the manufacturer’s recommended protocol. Two pads were attached to the patient’s thighs for grounding. The electrode needle was introduced into the tumor with CT guidance. The electrode tines were placed into the index tumor and deployed initially to 2 cm, and then–if needed–step-by-step to 3–5 cm. Time-at-temperature for each step varied according to the planned maximum deployment in agreement with the manufacturer’s guidelines. Before RFA, CT scans were obtained to ensure that all electrode tines were at suitable locations. A CT scan was performed immediately after RFA. To fully cover the index lesion with a 5-mm-ablative margin, overlapping RFAs would be performed if needed. Each lesion was ablated with one electrode needle. In every procedure, track RFA was performed before the electrode was withdrawn. Patients were routinely hospitalized for 2–3 days after RFA unless complications occurred.

### Assessments and Definitions

According to the previous study (27), tumor margins at portal venous and delayed phases on enhanced CT or MR imaging were categorized as: smooth margin, presenting as a simple nodular-shaped tumor that had a smooth tumor–normal liver parenchyma interface; non-smooth margin, presenting as tumor margin that had focal outgrowth of nodules or budding portion protruding into the nontumor parenchyma, or multi-nodular confluence appearance. The tumor margin was decided by the consensus of two diagnostic radiologists with 20 and 12 years of experience in abdominal imaging, respectively.

Complete ablation was defined as the absence of any enhancing lesion (indicating residual tumor) at the ablation site at contrast material–enhanced CT or MR imaging performed one month after RFA or TACE-RFA, which was decided by the consensus of the aforementioned two diagnostic radiologists. Ablative margin was defined as the margin of ablation zone beyond the border of the index tumor in order to achieve complete tumor destruction (28). It was classified into sufficient ablative margin (≥ 5 mm) and insufficient ablative margin (< 5 mm) referring to the previous study (28), using an evaluation based upon a two-screens slice-by-slice comparison of pre- and post-ablation CT or MR scans, which was decided by the aforementioned two diagnostic radiologists in consensus.

HCC recurrence was defined as the appearance of local tumor progression (LTP) or new HCC foci at follow-up contrast-enhanced CT or MR imaging after complete ablation was documented, which was decided by the aforementioned two diagnostic radiologists in consensus. According to the site of the first HCC recurrence, recurrence patterns were classified as follows: (a) LTP, which was defined as the appearance of enhancing tumor foci at the edge of the ablated zone (29); (b) intra-segmental recurrence, which was defined as any new emerging tumor that occurred in the liver separate from the ablated zone but within the same liver segment as the ablated zone according to Couinaud’s portal segmentation (30); (c) extra-segmental recurrence, which was defined as any new emerging tumor that occurred in different Couinaud’s segmentation from the ablated zone; and (d) aggressive recurrence, which was defined as the recurrence accompanied by portal vein or hepatic vein invasion, extrahepatic metastasis, or tumor seeding at the thoracic wall, abdominal wall, diaphragm, or peritoneal cavity. For patients with multiple recurrence patterns at first recurrence decision, the recurrence pattern of the patient was determined in the following order of priority: aggressive recurrence, extra-segmental recurrence, intra-segmental recurrence, and LTP. Besides, patient characteristics at HCC recurrent, including recurrent tumor size, recurrent tumor number, α-fetoprotein, and Child-Pugh grade, were documented.

### Follow-Up

All patients underwent follow-up one month after initial RFA or TACE-RFA. If complete ablation was not attained, the patient would undergo a second RFA. If complete ablation was achieved, follow-up was conducted every three months. The follow-up assessment included detailed history taking and physical examination, laboratory tests, an abdominal contrast-enhanced CT or MR examination, and chest CT scans. Laboratory tests included hematologic, biochemical analyses, and α-fetoprotein. Patients with clinical symptoms such as headache for brain metastasis or low back pain for spine metastasis underwent CT or MR examination for the detection of extrahepatic metastasis.

When HCC recurrences were recognized, salvage treatments were performed based on a consensus decision made by a multidisciplinary team, consisting of hepatobiliary surgeons, medical oncologists, interventional physicians, radiation therapists, and diagnostic radiologists. For patients with single or oligo (≤ 3) intrahepatic recurrent HCC, liver transplantation or resection, curative locoregional treatment (RFA, TACE-RFA, or percutaneous ethanol injection [PEI]), or TACE was performed. For patients with multiple (> 3) intrahepatic recurrent HCCs, TACE was performed. For patients with portal vein or hepatic vein invasion, tumor seeding, or extrahepatic metastasis, TACE, radiotherapy (external radiotherapy or 125I seeds brachytherapy), sorafenib, or conservative treatment was performed.

We compared the overall survival (OS) and post-recurrence survival among patients with different recurrence patterns. OS was defined as the time from the initial treatment until the date of death or the last follow-up. Post-recurrence survival was defined as the time from the first HCC recurrence until the date of death or the last follow-up. Patients who remained alive at the last follow-up were considered “censored” in statistical analysis. Patients who underwent liver transplantation or resection after recurrence were considered “censored” at the date of operation.

### Statistical Analysis

Categorical data were summarized as number of patients (percentage), and quantitative data were summarized as median value (range or interquartile range). The Pearson χ2 test and likelihood-ratio test were used to compare the categorical data between different recurrence patterns. The Mann-Whitney U test was used to compare the quantitative data between different recurrence patterns. The Cox proportional hazards regression model was used in the univariate analyses of risk factors for each recurrence pattern. In assessing the risk factors for each recurrence pattern, patients with other recurrence patterns were treated as censored data. Curves of OS and post-recurrence survival were determined by the Kaplan-Meier method, and the log-rank test was used for comparisons. Baseline characteristics and recurrence pattern were included in the univariate analyses of prognostic factors for OS, using the log-rank test. Variable with a *P*-value < 0.10 in the univariate analysis was included in the multivariate analysis. All multivariate analyses of prognostic factors were performed using Cox proportional hazards regression model. All tests were 2-sided, and *P* <.05 was considered statistically significant. All statistical analyses were performed using SPSS Statistics, version 19.0 (SPSS, Chicago, Il).

## Results

### Study Population

**Figure 1:**
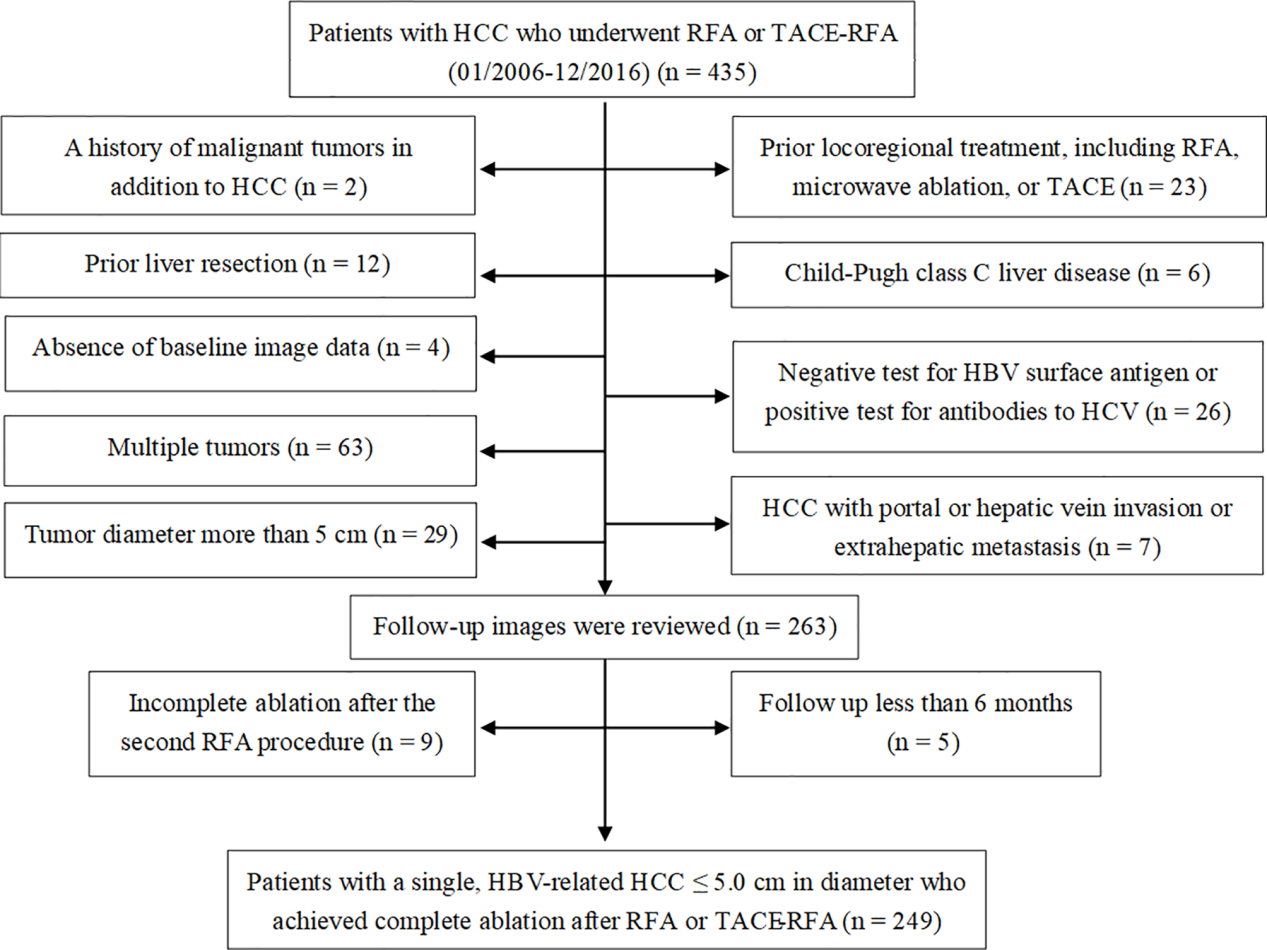
Flow diagram shows exclusion criteria in patients with hepatocellular carcinoma (HCC) who underwent radiofrequency ablation (RFA) or transarterial chemoembolization (TACE) combined with RFA (TACE-RFA). HBV, hepatitis B virus; HCV, hepatitis C virus.

A total of 435 patients with HCC underwent RFA or TACE-RFA during this study period. Among them, 186 patients were excluded from the study because they met the exclusion criteria (**Figure 1**). As a result, 249 patients with a single, HBV-related HCC ≤ 5.0 cm in diameter who achieved complete ablation after RFA or TACE-RFA were included in this study: 109 patients with HCC of ≤ 3.0 cm underwent RFA alone; 140 patients with HCC of 3.1-5.0 cm underwent TACE-RFA. Detailed patient characteristics are shown in **Table 1**.

**Table 1:** Baseline characteristics of the 249 patients.

Characteristic for patients	N = 249
**Sex (Male/Female)**	229 (92.0)/20 (8.0)
**Age (year), Median (range)**	55 (31–75)
**ECOG (0/1)**	228 (91.6)/21 (8.4)
**Child-Pugh Class (A/B)**	198 (79.5)/51 (20.5)
**HBV-DNA level (< 2000/≥ 2000 IU/mL)**	163 (65.5)/86 (34.5)
**HBeAg (Negative/Positive)**	189 (75.9)/60 (24.1)
**Tumor size (cm), Median (range)**	3.2 (0.8–5.0)
**Tumor size and treatment**	
** ≤ 2.0 cm with RFA alone**	26 (10.4)
** 2.1-3.0 cm with RFA alone**	83 (33.3)
** 3.1–5.0 cm with TACE-RFA**	140 (56.2)
**Tumor margin (Smooth/Non-smooth)**	143 (57.4)/106 (42.6)
**Subcapsular tumor (No/Yes)**	122 (49.0)/127 (51.0)
**Periportal tumor (No/Yes)**	199 (79.9)/50 (20.1)
**Perivenous tumor (No/Yes)**	203 (81.5)/46 (18.5)
**α-fetoprotein (ng/mL), Median (IQR)**	46.2 (9.7–78.3)
**α-fetoprotein (< 100/≥ 100 ng/mL)**	207 (83.1)/42 (16.9)
**Total bilirubin (umol/L), Median (IQR)**	15 (10.9–22)
**Serum albumin (g/dL), Median (IQR)**	39.2 (36–43.7)
**Platelet (10^9^/L), Median (IQR)**	128 (90–159.5)
**RFA procedures to complete ablation (1/2)**	238 (95.6)/11 (4.4)
**Ablative margin (Sufficient/Insufficient/NA)**	83 (33.3)/160 (64.3)/6 (2.4)

Except where indicated, data are numbers of patients, numbers in parentheses are percentages. IQR, interquartile range; ECOG, Eastern Cooperative Oncology Group; HBV, hepatitis B virus; RFA, radiofrequency ablation, TACE-RFA, transarterial chemoembolization combined with radiofrequency ablation, NA, not available.

There was no treatment-related mortality. Major complications occurred in five patients (2.0%) and were successfully managed, including one case each of left heart failure, biloma, and acute extensive portal vein thrombosis, and two cases of liver abscess. Minor complications occurred in 27 patients (10.8%). Details of complications were shown in **Table 2**.

**Table 2:** Complications of radiofrequency ablation with or without transarterial chemoembolization (N = 249).

Complications	Patients
**Major complications**	5 (2.0)
Left heart failure^*^	1 (0.4)
**Acute extensive portal vein thrombosis^#^**	1 (0.4)
**Biloma^&^**	1 (0.4)
**Liver abscess^&^**	2 (0.8)
**Minor complications**	27 (10.8)
**Arterial-portal vein fistula**	3 (1.2)
**Hepatic segment infarction**	4 (1.6)
**Portal vein branch stricture**	3 (1.2)
**Segmental bile duct dilatation**	3 (1.2)
**Subcapsular hemorrhage**	7 (2.8)
**Pleural effusion**	5 (2.0)
**Jaundice^†^**	2 (0.8)
**Total**	32 (12.8)

Except where indicated, data are numbers of patients, numbers in parentheses are percentages.

^*^Left heart failure was cured two days after constant oxygen inhalation, vasodilator, cardiotonic therapy and diuretic treatment.

^#^The portal vein thrombosis achieved complete resolution 14 days after anticoagulant therapy.

^&^Biloma and liver abscesses were successfully managed by percutaneous puncture and draining and use of antibiotics.

**^†^**This complication means there was a transient increase in serum total bilirubin level of more than 3 mg/dL (51.3 mmol/L) after radiofrequency ablation without bile duct injury.

### Recurrence Patterns and Salvage Treatments

The median follow-up was 53 months (range, 7-114 months). During the follow-up, 163 of the 249 patients (65.5%) experienced HCC recurrence. The 1-, 3-, and 5-year cumulative recurrence rates were 21.3%, 59.7%, and 72.6%, respectively. The recurrence patterns included LTP in 40 patients (16.1%), intra-segmental recurrence in 43 patients (17.3%), extra-segmental recurrence in 62 patients (24.9%), and aggressive recurrence in 18 patients (7.2%).

Characteristics of recurrent tumors of each pattern were shown in **Table 3**. Patients with extra-segmental recurrence had a longer time to recurrence (median, 28.5 months) than patients with other recurrence patterns (median, 11-14 months) (*P* < 0.001).

**Table 3:** Characteristics, salvage treatments, and outcomes for different recurrence patterns.

Characteristic	LTP (n = 40)	Intra-segmental recurrence (n = 43)	Extra-segmental recurrence (n = 62)	Aggressive recurrence^*^ (n = 18)
**Time to recurrence (month)**				
**Median (range)**	11 (5-35)	14 (4-39)	28.5 (5-55)	12 (3-45)
**Recurrent tumor size (cm)**				
**Median (range)**	1.3 (0.8-3.6)	1.6 (0.8-3)	1.5 (0.8-2.6)	2.0 (0.8-5.6)
**Recurrent tumor number**				
**Solitary/Multiple**	40 (100)/0	36 (83.7)/6 (14)	46 (74.2)/16 (25.8)	8 (44.4)/10 (55.6)
**Salvage treatment**				
**Surgery^†^**	2 (5.0)	3 (7.0)	1 (1.6)	0
**Curative locoregional treatment^#^**	36 (90.0)	34 (79.1)	42 (67.8)	0
**TACE**	2 (5.0)	5 (11.6)	14 (22.6)	3 (16.7)
**Others^&^**	0	1 (2.3)	5 (8.1)	15 (83.3)
**OS (month), Median (95% CI)**	65 (57.1-72.9)	56 (52.1–59.9)	58 (52.6–63.4)	28 (17.6–38.4)
**Post-recurrence survival (month), Median (95% CI)**	49 (40.2–57.8)	37 (28.3–45.7)	25 (20.6–29.3)	15 (11.1–18.9)

Except where indicated, data are numbers of patients, numbers in parentheses are percentages. LTP, local tumor progression; OS, overall survival; CI, confidence interval. *Aggressive recurrence included extrahepatic metastasis in 10 patients, portal vein invasion in 4 patients, hepatic vein invasion in 2 patients, and abdominal wall tumor seedings in 2 patients. ^†^Including liver transplantation or resection. ^#^Including radiofrequency ablation, transarterial chemoembolization combined with radiofrequency ablation, or percutaneous ethanol injection. ^&^Including radiotherapy, sorafenib, or conservative treatment.

Among these 163 patients, 6 underwent liver transplantation or resection, 112 underwent RFA, TACE-RFA or PEI, 24 underwent TACE, and 21 underwent other treatments, including radiotherapy, sorafenib, or conservative treatment.

### Risk Factors of Each Recurrence Pattern

The results of univariate and multivariate analyses for risk factors of each recurrence pattern were shown in **Table 4**. The results revealed that: tumor sized 2.1-3.0 cm undergoing RFA alone (vs. tumor sized 3.1-5.0 cm undergoing TACE-RFA, HR [95% CI]: 2.374 [1.229, 4.588], *P* = 0.010) and insufficient ablative margin (HR [95% CI]: 4.521 [1.737–11.769], *P* = 0.002) were independent risk factors for LTP; a periportal tumor (HR [95% CI]: 2.361 [1.245, 4.478], *P* = 0.009) and non-smooth tumor margin (HR [95% CI]: 2.380 [1.297, 4.369], *P* = 0.005) were independent risk factors for intra-segmental recurrence; HBV-DNA level ≥ 2000 IU/mL (HR [95% CI]: 2.306 [1.396–3.811], *P* = 0.001) was an independent risk factor for extra-segmental recurrence; periportal tumor (HR [95% CI]: 3.896 [1.529–9.930], *P* = 0.004) and α-fetoprotein level ≥ 100 ng/mL (HR [95% CI]: 2.924 [1.120–7.633], *P* = 0.028) were independent risk factors for aggressive recurrence.

**Table 4:** Univariate and multivariate analysis of the risk factors for different recurrence patterns.

Factors	Univariate analysis		Multivariate analysis	
	HR (95% CI)	*P* value	HR (95% CI)	*P* value
**Local tumor progression**				
**Gender**		.041		.181
**Male**	1		…	
** Female**	2.339 (1.034–5.288)		…	
**Tumor size and treatment**		.047		.031
** 3.1–5.0 cm with TACE-RFA**	1		1	
** ≤ 2.0 cm with RFA alone**	.536 (.124–2.312)	.403	.897 (.204–3.940)	.885
** 2.1-3.0 cm with RFA alone**	1.961 (1.037–3.707)	.038	2.374 (1.229–4.588)	.010
**Tumor margin**		.016		.055
**Smooth**	1		…	
**Non-smooth**	2.159 (1.152–4.045)		…	
**Ablative margin**		.003		.002
** Sufficient**	1		1	
** Insufficient**	4.150 (1.619–10.636)		4.521 (1.737–11.769)	
**Intra-segmental recurrence**				
**Tumor size and treatment**		.065		.115
** 3.1–5.0 cm with TACE-RFA**	1		…	
** ≤ 2.0 cm with RFA alone**	.437 (.134–1.426)	.437	…	
** 2.1-3.0 cm with RFA alone**	.443 (.204–.962)	.040	…	
**Periportal tumor**		.009		.009
**No**	1		1	
**Yes**	2.345 (1.236–4.449)		2.361 (1.245–4.478)	
**Tumor margin**		.005		.005
**smooth**	1		1	
** non-smooth**	2.369 (1.291–4.349)		2.380 (1.297–4.369)	
**Extra-segmental recurrence**				
**Child-Pugh class**		.084		.168
** A**	1		…	
** B**	1.683 (.932–3.037)		…	
**HBV-DNA level**		.001		.001
** < 2000 IU/mL**	1		1	
** ≥ 2000 IU/mL**	2.306 (1.396–3.811)		2.306 (1.396–3.811)	
**Aggressive recurrence**				
**Perivenous tumor**		.077		.170
**No**	1		…	
**Yes**	2.420 (.908–6.448)		…	
**Periportal tumor**		.002		.004
**No**	1		1	
**Yes**	4.400 (1.743–11.109)		3.896 (1.529–9.930)	
**α-fetoprotein level**		.010		.028
** < 100 ng/mL**	1		1	
** ≥ 100 ng/mL**	3.485 (1.347–9.016)		2.924 (1.120–7.633)	

HR, hazard ratio; CI, confidence interval; RFA, radiofrequency ablation, TACE-RFA, transarterial chemoembolization combined with radiofrequency ablation; HBV, hepatitis B virus.

### OS and Prognostic Factors

**Figure 2:**
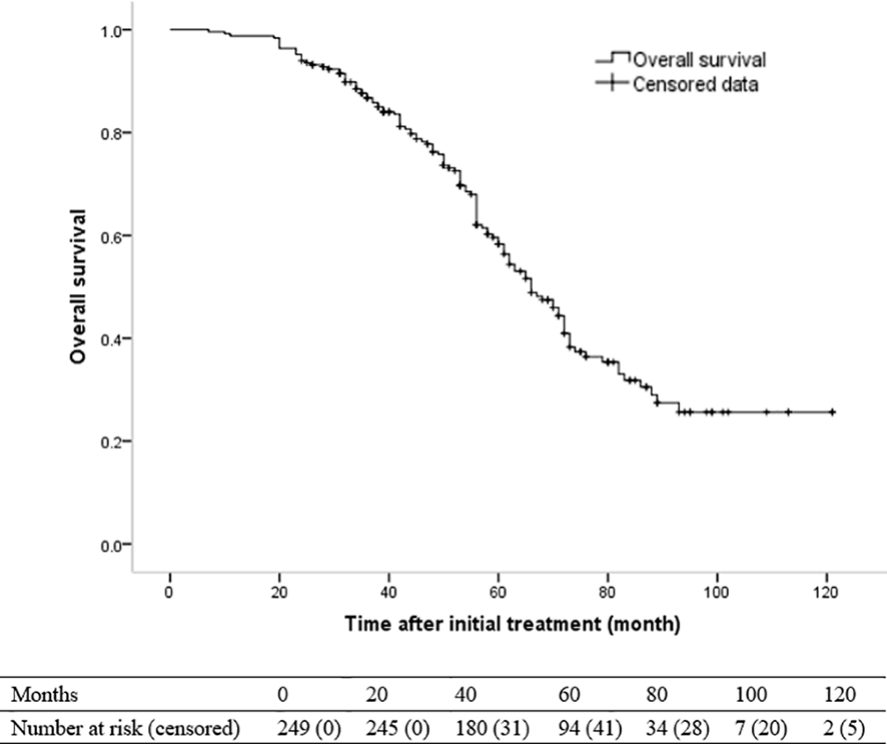
Kaplan-Meier curve of overall survival (OS) in patients with hepatocellular carcinoma who underwent radiofrequency ablation with or without transarterial chemoembolization (median OS, 66 months).

One hundred and twenty-two patients died during the follow-up. The 1-, 3- and 5-year OS rates were 98.8%, 86.7% and 58.3%, respectively. The median OS was 66 months (95% CI, 59.8–72.2 months) (**Figure 2**). The univariate and multivariate analyses revealed that Child-Pugh class B (HR [95%]: 1.653 [1.066–2.563], *P* = 0.025) and recurrence pattern (HR [95% CI] of each pattern when compared with LTP: intra-segmental recurrence, 1.618 [.948–2.763]; extra-segmental recurrence, 1.822 [1.064–3.120]; aggressive recurrence, 9.363 [4.845–18.094]; No recurrence, 238 [.122–.467]; overall *P < *0.001) were independent prognostic factors for OS (**Table 5**).

**Table 5:** Univariate and multivariate analysis of the prognostic factors for overall survival in the entire study population (N = 249).

Factors	OS (month), Median (95% CI)	*P* value	HR (95% CI)	*P* value
**Child-Pugh Class**		.002		.025
** A**	71 (66.2–75.7)		1	
**B**	58 (54.2–61.7)		1.653 (1.066–2.563)	
**Tumor margin**		.078		.681
** Smooth**	72 (66.7–77.3)		…	
** Non-smooth**	62 (57.8–66.2)		…	
**HBV-DNA level**		.003		.065
** < 2000 IU/mL**	71 (65.2–76.8)		…	
** ≥ 2000 IU/mL**	56 (53.1–58.9)		…	
**α-fetoprotein level**		.054		.425
** < 100 ng/mL**	70 (65.2–74.8)		…	
** ≥ 100 ng/mL**	61 (45.3–76.6)		…	
**Ablative margin**		.076		.072
** Sufficient**	71 (63.5–78.5)		…	
**Insufficient**	62 (55.1–68.9)		…	
**Recurrence pattern**		<.001		<.001
** LTP**	65 (57.1–72.9)		1	
** Intra-segmental recurrence**	56 (52.1–59.9)	.053	1.618 (.948–2.763)	.078
** Extra-segmental recurrence**	58 (52.6–63.4)	.022	1.822 (1.064–3.120)	.029
** Aggressive recurrence**	28 (13.4–42.5)	<.001	9.363 (4.845–18.094)	<.001
** No recurrence**	> 86^*^	<.001	.238 (.122–.467)	<.001

OS, overall survival; CI, confidence interval; HR, hazard ratio; HBV, hepatitis B virus; LTP, local tumor progression.

^*^The median OS for patients with no recurrence was not reached, and the estimated OS rate was 60.7% at 86 months using Kaplan-Meier method.

**Figure 3:**
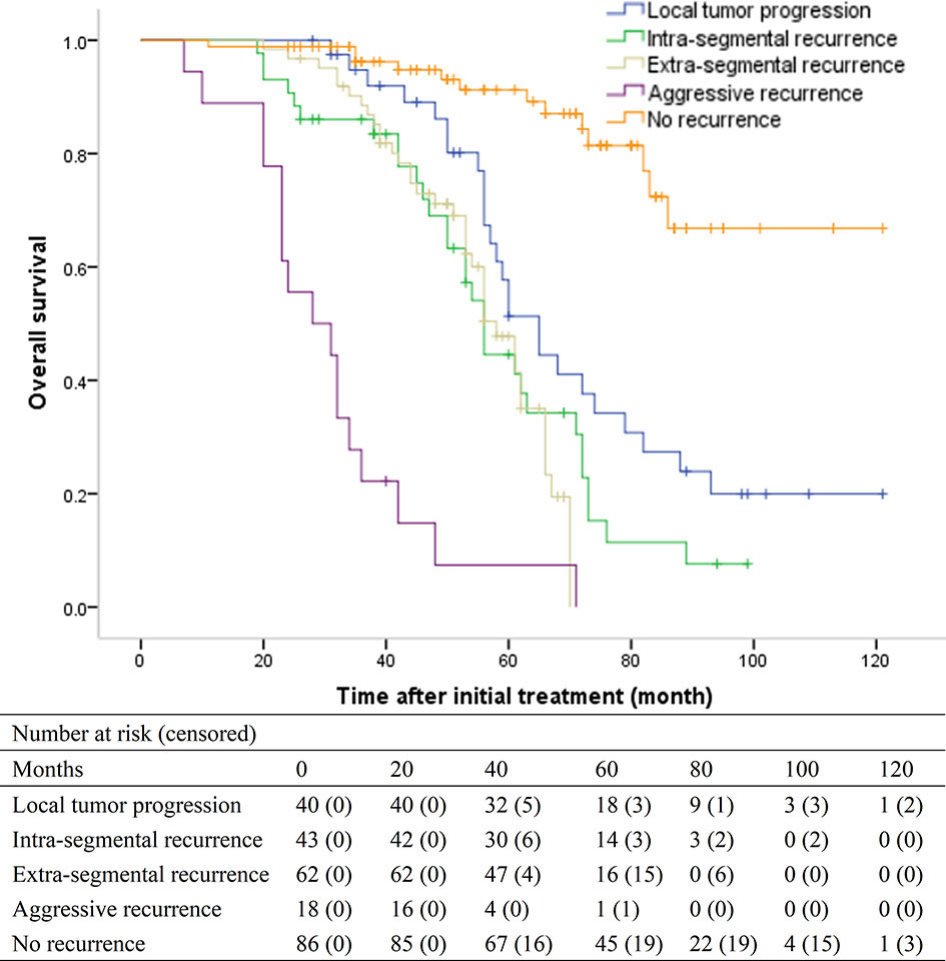
Kaplan-Meier curves of overall survival (OS) in patients with local tumor progression (median, 65 months), intra-segmental recurrence (median, 56 months), extra-segmental recurrence (median, 58 months), aggressive recurrence (median, 28 months), and no recurrence (median, > 86 months), respectively (overall *P* <.001).

For patients with LTP, intra-segmental recurrence, extra-segmental recurrence, aggressive recurrence, and no recurrence, the median OS was 65 months (95% CI, 57.1-72.9 months), 56 months (95% CI, 52.1–59.9), 58 months (95% CI, 52.6–63.4), 28 months (95% CI, 17.6–38.4), and > 86 months, respectively (overall *P* < .001) (**Figure 3**). Patients with LTP had the best OS among the four recurrence patterns. Patients with aggressive recurrence had the worst OS. However, patients with intra-segmental recurrence had similar OS with patients with extra-segmental recurrence (*P* = .470).

### Post-Recurrence Survival

**Figure 4:**
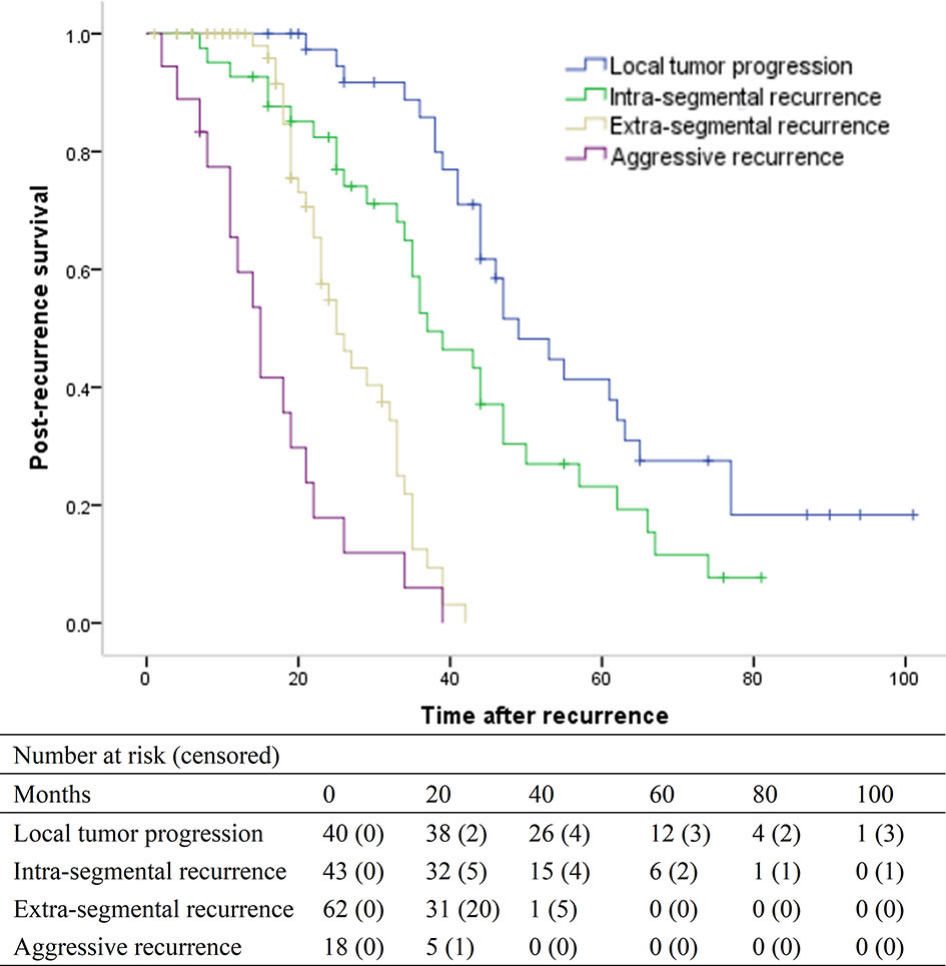
Kaplan-Meier curves of post-recurrence survival in patients with local tumor progression (median, 49 months), intra-segmental recurrence (median, 37 months), extra-segmental recurrence (median, 25 months), and aggressive recurrence (median, 15 months), respectively (overall *P* <.001).

For the 163 patients with recurrence, the median post-recurrence survival was 35 months (95% CI, 32.5–37.5 months). For patients with LTP, intra-segmental recurrence, extra-segmental recurrence, and aggressive recurrence, the median post-recurrence survival was 49 months (95% CI, 40.2–57.8), 37 months (95% CI, 28.3–45.7), 25 months (95% CI, 20.6–29.3), and 15 months (95% CI, 11.1–18.9), respectively (overall *P* <.001) (**Figure 4**). The median post-recurrence survival for patients with intra-segmental recurrence was better than that for patients with extra-segmental recurrence (*P* < 0.001).

## Discussion

Our study classified HCC recurrence after RFA or TACE-RFA into four patterns: LTP, intra-segmental recurrence, extra-segmental recurrence, and aggressive recurrence; and the median OS among patients with different patterns showed markedly difference as follows: LTP (65 months) > intra-segmental (56 months) or extra-segmental recurrence (58 months) > aggressive recurrence (28 months). Although the median OS did not have significant difference between patients with intra-segmental recurrence and patients with extra-segmental recurrence, the former had a shorter time to recurrence (median, 14 months vs. 28.5 months) and a longer post-recurrence survival (median, 37 months vs. 25 months). Additionally, we found that the recurrence pattern was an independent prognostic factor for OS in uni- and multivariable analyses, and each recurrence pattern had different risk factors. These results implied the usefulness of this classification for HCC recurrence, which provides us an important clinical reference value to take preventive measures for each recurrence pattern according to these risk factors, and helps us predict the long-term survival of recurrence patients. Our study also found that Child-Pugh class B was another independent predictor for OS, which was similar to previous studies reported (17, 22). Child-Pugh class B liver disease may cause intolerance of repeated salvage treatment or even liver failure during a long-term follow-up.

Our study found that patients with LTP had the best OS among patients with HCC recurrence, which may be attributed to curative salvage treatments performed in 95% patients with LTP. These patients had a small recurrent tumor size (median, 1.3 cm). Our study further confirmed the results of previous studies: to reduce LTP, TACE-RFA should be suggested for HCC sized 3.1-5.0 cm (9–11); RFA alone is appropriate for HCC sized ≤ 2.0 cm (2, 31). However, whether TACE-RFA can reduce LTP in patients with HCC sized 2.1-3.0 cm remains controversial (16, 17, 32, 33). Our results revealed that tumor sized 2.1-3.0 cm undergoing RFA alone was an independent risk factor for LTP. We speculate that this result may be due to a high proportion (55.4%) of HCC at special location (subcapsular, periportal, or perivenous). In previous studies, for patients with HCC sized 2.1-3.0 cm (32, 33), especially those at special location (34), TACE-RFA may improve the local tumor control. Therefore, we suggest that TACE-RFA should be considered in HCC sized 2.1-3.0 cm at special location. The ablative margin was another important risk factor for LTP in our study. Previous study reported that imaging-invisible micro-metastasis might surround HCC (35). In order to completely ablate these microsatellite foci, a 5-mm-ablative margin is usually needed (28).

In previous studies (8, 21), a new emerging tumor in the liver but separate from the ablated area was defined as intrahepatic distant recurrence. We further classified these recurrences into intra-segmental recurrence and extra-segmental recurrence. In fact, we confirmed that patients with extra-segmental recurrence had a longer median time to recurrence (28.5 vs. 14 months) than patients with intra-segmental recurrence. The longer time to recurrence contributed to the OS in patients with extra-segmental recurrence. Moreover, we further confirmed that patients with extra-segmental recurrence had a shorter post-recurrence survival (median, 14 months vs. 28.5 months) than patients with intra-segmental recurrence. Both the time to recurrence and post-recurrence survival resulted in a similar OS between intra-segmental and extra-segmental recurrence (56 months vs. 58 months).

Intra-segmental recurrences could be controlled effectively by salvage RFA or TACE since these tumors were limited in the same liver segment. Our study showed that periportal tumor and non-smooth tumor margin were independent risk factors for intra-segmental recurrence. A periportal location increases the risk of dissemination of HCC through the portal system (12). Besides, a non-smooth tumor margin on imaging was reported to be associated with microvascular invasion in histopathologic studies (27), and it may cause early HCC recurrence by tumor spread after curative treatment (18, 27).

The extra-segmental recurrence has a broader range of liver involved by HCC, which conducts to the difficulty of salvage treatment. In fact, our study confirmed that extra-segmental recurrence had a higher proportion of multiple recurrent tumors than intra-segmental recurrence. The extra-segmental recurrence may be derived from both *de novo* carcinogenesis and intrahepatic tumor spread. Our results showed that HBV-DNA level ≥ 2000 IU/mL was an independent risk factor for extra-segmental recurrence. It was reported that a high HBV-DNA level increase HCC recurrence by *de novo* carcinogenesis arising from repeated destruction and regeneration in the inflammatory liver parenchyma (15). Moreover, a high HBV load was reported to promote HCC growth, invasiveness, and metastasis (36).

Previous studies (19, 22) classified extrahepatic metastasis solely as one recurrence pattern. Considering that patients with portal or hepatic vein tumor invasion or tumor seeding are at an advanced stage according to the Barcelona Clinic Liver Cancer staging system and have poor survival outcomes, we defined aggressive pattern including these recurrences and extrahepatic metastasis. The result showed that these patients with aggressive recurrence had the worst OS. The independent predictors for aggressive recurrence were periportal tumor and α-fetoprotein level ≥ 100 ng/mL in our study. The heat-sink effect of the portal vein during RFA could lead to residual tumor cells in patients with periportal HCC (37). These residual HCC cells may transform into a more aggressive cellular phenotype after going through sublethal temperatures (14) and accelerate HCC recurrence. Besides, after performing RFA for a periportal HCC, there was an increasing risk of extrahepatic spread (20) through the portal system or rapid intrahepatic neoplastic progression even complete local necrosis was achieved (12, 38). A high α-fetoprotein level indicates poor differentiation and high aggressiveness of the initial HCC, which was also associated with neoplastic progression after treatment (13, 21).

There are several limitations of our study: First, it was a single-center retrospective study, and thus was inherently flawed by selection and indication bias; Second, in majority of the cases (83%), there was no pathological diagnosis; Third, we evaluated ablative margin by using a slice-by-slice comparison method, which was not as accurate as an image fusion method. However, this method was practical in evaluating a 5-mm-ablative margin, and the ablative margin was shown clearly in tumors with lipiodol retention of TACE. The conclusions of this study need to be verified by prospective studies with larger sample size.

In conclusion, HCC recurrence patterns after RFA or TACE-RFA could be classified into LTP, intra-segmental recurrence, extra-segmental recurrence, and aggressive recurrence. For different recurrence patterns, there were different risk factors, OS, and post-survival survival. Recurrence pattern and Child-Pugh class B were independent predictors for OS.

## Data Availability Statement

The raw data supporting the conclusions of this article will be made available by the authors, without undue reservation.

## Ethics Statement

The studies involving human participants were reviewed and approved by Institutional review board of Second Affiliated Hospital of Guangzhou Medical University. Written informed consent for participation was not required for this study in accordance with the national legislation and the institutional requirements.

## Author Contributions

JH: conceptualization, methodology, validation, investigation, data curation, writing—original draft preparation, and writing—review and editing. WH: conceptualization, methodology, validation, investigation, resources, data curation, writing—original draft preparation, and writing—review and editing. YG: conceptualization, methodology, validation, and investigation. MC: methodology, formal analysis, investigation, data curation, and writing—review and editing. JZ: investigation, writing—original draft preparation, and writing—review and editing. LL: investigation and writing—original draft preparation. KZ: conceptualization, writing—review and editing, supervision, project administration, and funding acquisition. All authors contributed to the article and approved the submitted version.

## Funding

This work was supported by the National Natural Science Foundation of China (No. 81873920, 82001930), Medical Scientific Research Foundation of Guangdong Province, China (No. B2019055), and High-Level University Clinical Research Promotion Program of Guangzhou Medical University (No. B185004019).

## Conflict of Interest

The authors declare that the research was conducted in the absence of any commercial or financial relationships that could be construed as a potential conflict of interest.
